# Saffron: A Look from the Customer in Vietnam

**DOI:** 10.21315/mjms2023.30.2.17

**Published:** 2023-04-18

**Authors:** Le Pham Tan Quoc

**Affiliations:** Institute of Biotechnology and Food Technology, Industrial University of Ho Chi Minh City, Ho Chi Minh City, Vietnam

Dear Editor,

I read with interest the manuscript titled ‘A systematic review on the effect of saffron extract on lipid profile in hyperlipidaemic experimental animal models’ by Abd Rahim et al. (1). In fact, saffron possesses many medicinal properties because it contains a large number of bioactive compounds, especially carotenoids (crocin and crocetin) and terpenes (2). Some previous reports also revealed that this material plays the role of an antioxidant, anti-inflammatory, antinociceptive, antidepressant, antitussive, anticonvulsant, memory enhancer, hypotensive and anticancer (3). Although more and more new positive properties of saffron have been discovered, almost all of these experiments were conducted in animals, especially in mice and data is limited in humans, with only a few clinically relevant studies. Thus, we have to wait a long time to affirm the final conclusion about the medical properties of saffron.

Saffron has been considered as a food for humans (colourant and flavouring agent, tea, spice, etc.) in daily meals. For medical and pharmaceutical purposes, some studies use ethanol, aqueous ethanol and water to isolate bioactive compounds from raw materials (1). We can also use methanol, ethyl acetate and other solvents to recover the chemical components. These compounds can be completely different from the compositions in an ethanol/water extract as they depend on the polarity of the solvent. Maybe the obtained compounds have the special characteristics that we need.

In general, the quality of saffron depends on the content and efficacy of crocin; however, the consumer is unaware of this. Under the inflated effect of the media in Vietnam, the daily use of saffron has become trendy, although the price is quite expensive (approximately USD20 per gram). However, fake saffron has appeared and spread everywhere, made from corn silk threads, dried beef/pork yarn and coconut filaments. Fortunately, these materials originate from food and do not significantly affect human health. In some cases, dyed horse hair or shredded paper has been used to replace saffron, especially to enable a cheaper price. These fake materials will be dyed with synthetic colour, which is harmful to health.

Note that saffron exhibits no significant toxicity in both clinical and experimental investigations (acute, sub-acute, sub-chronic and chronic studies). However, using a high dose of saffron can cause teratogenic effects in mice (4) and we are therefore careful when using it for pregnant women, although there are not any recommendations on this subject. Hariri et al. (5) argued that the presence of safranal at high doses in saffron can induce genotoxicity. In fact, the advantages and disadvantages of saffron are still controversial; however, until now, its strong points seem to dominate.

In general, saffron possesses many areas of interest that remain to be discovered in terms of medicine and nutrients. However, let’s be intelligent customers; we should buy it from reputable companies and research carefully, to avoid buying fake and poor-quality goods on e-commerce platforms. Moreover, to protect ourselves, we should not consider saffron as an elixir and abuse it.

## References

[1] Abd Rahim IN, Mohd Kasim NA, Isa MR, Nawawi H (2022). A systematic review on the effect of saffron extract on lipid profile in hyperlipidaemic experimental animal models. Malays J Med Sci.

[2] Midaoui AE, Ghzaiel I, Vervandier-Fasseur D, Ksila M, Zarrouk A, Nury T (2022). Saffron (*Crocus sativus* L.): a source of nutrients for health and for the treatment of neuropsychiatric and age-related diseases. Nutrients.

[3] Razavi BM, Hosseinzadeh H (2015). Saffron as an antidote or a protective agent against natural or chemical toxicities. Daru J Pharm Sci.

[4] Al-Qudsi F, Ayedh A (2012). Effect of saffron on mouse embryo development. J Am Sci.

[5] Hariri AT, Moallem SA, Mahmoudi M, Hosseinzadeh H (2011). The effect of crocin and safranal, constituents of saffron, against subacute effect of diazinon on hematological and genotoxicity indices in rats. Phytomedicine.

